# Role of dopamine signaling in male courtship suppression induced by confinement stress in *Drosophila*

**DOI:** 10.1016/j.isci.2026.115906

**Published:** 2026-04-27

**Authors:** Tomohito Sato, Rana Toyama, Toshihiro Kitamoto, Takaomi Sakai

**Affiliations:** 1 Department of Biological Sciences, Tokyo Metropolitan University, 1-1 Minamiosawa, Hachioji, Tokyo 192-0397, Japan; 2 Department of Anesthesia and Neuroscience & Pharmacology, Carver College of Medicine, University of Iowa, Iowa City, IA 52242, USA; 3 Interdisciplinary Graduate Programs in Genetics and Neuroscience, University of Iowa, Iowa City, IA 52242, USA

**Keywords:** Biological sciences, Neuroscience, Behavioral neuroscience, Molecular neuroscience

## Abstract

Stress disturbs physiological and psychological homeostasis across species. In mammals, stress reduces male courtship motivation, but the underlying neuronal mechanisms remain poorly understood. Here, we establish a *Drosophila* model in which confinement to a small space without complete immobilization—termed small-space (SS) stress—suppresses male courtship behavior. Because stress modulates dopamine signaling in both vertebrates and invertebrates, we examined its role in SS-stress-induced courtship suppression. Pharmacological inhibition and genetic manipulations revealed that dopamine synthesis, release, and reception are required to maintain—but not initiate—the SS-stress-induced suppression of male courtship. Furthermore, dopamine release to and reception within the mushroom body—a brain region involved in higher-order sensory processing—were essential for sustaining courtship inhibition after stress. This SS stress paradigm provides a robust framework for elucidating dopamine-mediated mechanisms that support persistent behavioral changes after stress and contribute to a deeper understanding of the neurobiological basis of stress-related sexual dysfunction.

## Highlights

• *Drosophila* males show courtship suppression after confinement in a small space
• The duration of courtship suppression correlates with the stress length
• Dopamine signaling is required to sustain stress-induced courtship suppression
• Dopamine-responsive MB neurons are involved in sustaining courtship suppression

## Graphical abstract

**Figure:**
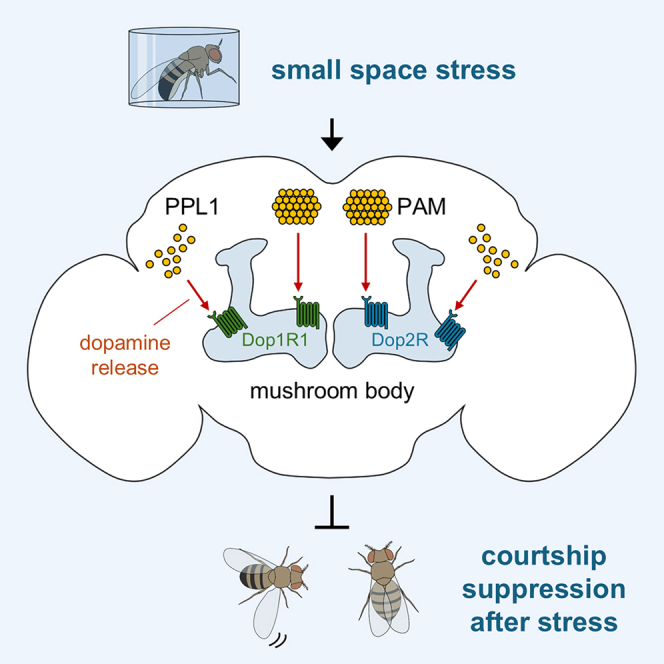

## Introduction

Stress—whether arising from external or internal stimuli—can significantly disrupt an animal’s physiological and psychological balance, leading to alterations in brain function and overall homeostasis. Exposure to stressful conditions induces lasting plastic changes in the brain that can affect both physiology and behavior after the stress experience ended.^1^ Across species, including humans, both acute and chronic stressors affect a wide range of behavioral outputs, such as the wake-sleep cycle,^2^ emotional behavior,^3^^,^^4^ escape responses,^5^^,^^6^ locomotion,^7^^,^^8^ and feeding patterns.^9^^,^^10^

Under stress, neurotransmitter release and signaling dynamics are altered, leading to diverse physiological and behavioral outcomes.^11^ Among these transmitters, dopamine plays a particularly important role in these processes. In rodents and humans, stress can either enhance or suppress dopaminergic function, depending on its type, duration, and intensity.^12^ Interestingly, stress-induced modifications of dopaminergic function are evolutionarily conserved. For instance, mechanical stress causes a 4-fold increase in dopamine levels in the hemolymph of oysters.^13^ In the fruitfly *Drosophila*, dopamine levels rise following heat stress,^14^^,^^15^ and mechanical stress increases the enzymatic activity of tyrosine hydroxylase (TH), the rate-limiting enzyme for dopamine synthesis, whereas starvation stress decreases TH activity.^16^ Similarly, stress exposure reduces dopamine levels in ants and bees.^17^^,^^18^ Collectively, these findings underscore the widespread effects of stress on dopaminergic function across both vertebrates and invertebrate species.

Sexual behavior, like other intrinsic behaviors, is also highly sensitive to stress. In humans, individuals with post-traumatic stress disorder often exhibit sexual dysfunction.^19^ Likewise, male rats show reduced sexual motivation after exposure to stressors such as foot shock or predator odors.^20^^,^^21^ Despite these observations, the molecular and cellular mechanisms by which stress diminishes sexual motivation remain poorly understood.

*Drosophila melanogaster* serves as a powerful model system for dissecting the neuronal mechanisms linking stress to behavior, owing to its extensive neurogenetic toolkit and well-characterized behavioral paradigms.^7^^,^^22^ *Drosophila* has also long been used to study sexual behavior,^23^^,^^24^^,^^25^ and recent advances have led to the identification of specific neuronal circuits and molecular pathways that regulate mating drive and sexual motivation.^26^^,^^27^^,^^28^ Notably, dopamine neurons and dopamine release play critical roles in controlling male mating drive in *Drosophila*.^29^^,^^30^ Thus, this model organism provides a unique opportunity to explore how stress modulates sexual motivation and to define the role of dopamine in this process.

Confinement in a small space is stressful for many animals. However, the neuronal mechanisms by which confinement stress with movement restriction affects brain function and physiology remain poorly understood. In this study, we established a *Drosophila* stress paradigm by confining individual males in very small acrylic chambers that limit movement without completely immobilizing the body—referred to as small-space (SS) stress. Under this condition, flies cannot walk freely but can still move their legs and rotate their bodies, distinguishing this paradigm from the full restraint stress methods commonly used in rodents. Using this approach, we found that SS stress significantly suppresses male courtship activity toward virgin females. Furthermore, we demonstrated that dopamine signaling is crucial for mediating this stress-induced suppression of male courtship activity.

## Results

### SS confinement induces transient and duration-dependent suppression of male courtship behavior

**Figure 1:**
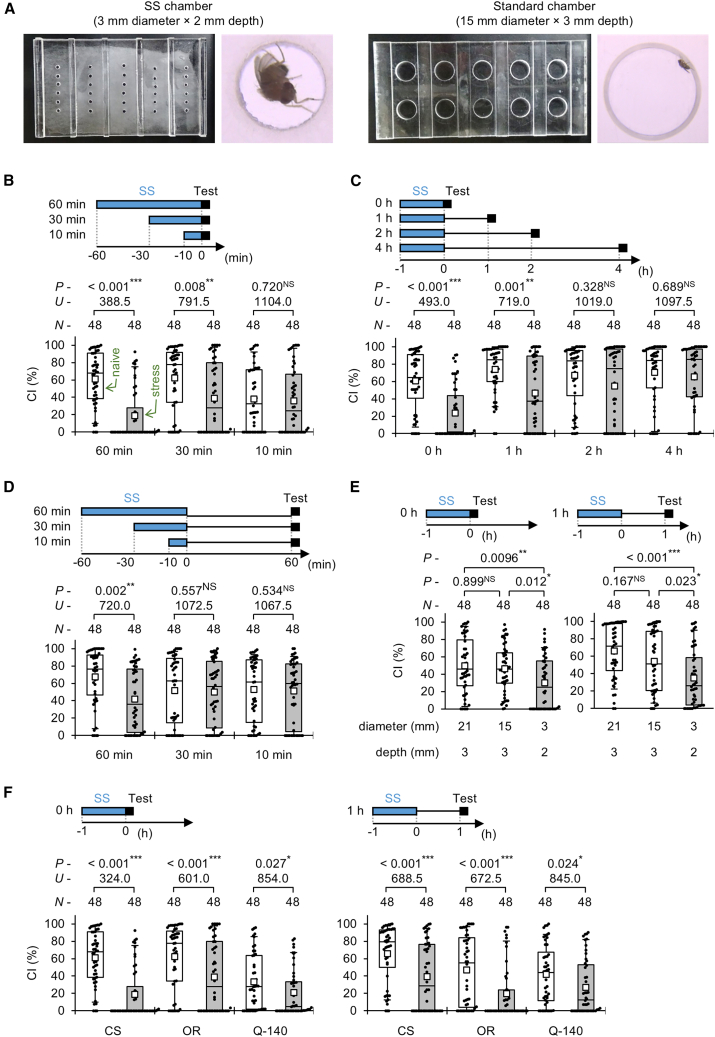
Male courtship suppression after the experience of SS stress (A) Single males were introduced into the SS chamber to expose them to SS stress (left). Naive males introduced into the standard chamber were used as a control (right). (B) Male courtship behavior was observed after SS stress in each experiment. Single intact virgin male and freeze-killed virgin female couples were transferred into observation chambers (15 mm diameter, 3 mm depth), and courtship behaviors were videotaped for 10 min. For more details, see STAR Methods. In the test, courtship activity was measured immediately after 60-, 30-, and 10-min SS stress. Wild-type (CS) males were used in the experiments. (C) Courtship activity was measured immediately, 1 h, 2 h, and 4 h after 1-h SS stress. CS males were used in the experiments. (D) Male flies were confined in SS chambers for either 60, 30, or 10 min. Behavioral tests were carried out 1 h after each confinement. (E) CS males were confined for 1 h using three types of chambers as follows: Large chamber (21 mm in diameter, 3 mm in depth), standard chamber (15 mm in diameter, 3 mm in depth), and SS chamber (3 mm in diameter, 2 mm in depth). Male courtship behavior was observed immediately and 1 h after the confinement in each chamber. (F) Courtship activity in three wild-type strains (CS, OR, and Q-140) was measured immediately (0 h) and 1 h after 1-h SS stress. (B–F) We visualized the data using a boxplot with individual data points (black dots). In each graph, white boxes indicate naive males, and gray boxes indicate stressed males. Boxplots for a set of CI data show the 10th, 25th, 75th, and 90th centiles. In the boxplots, white squares indicate the mean, and the lines are drawn at the median. For statistical comparisons, the Mann-Whitney *U* test was used for CI, except for figure E. For the multiple comparison in figure (E), the Kruskal-Wallis test followed by the Steel-Dwass test was used. *n*, sample size; ∗∗∗, *p* < 0.001; ∗∗, *p* < 0.01; ∗, *p* < 0.05; NS, not significant. See also Figures S1 and S2.

To expose flies to SS stress, sexually mature virgin males (4–6 days old) of the wild-type strain Canton-S (CS) were cold-anesthetized and then placed in a tiny chamber, where they were confined for 10, 30, and 60 min (Figure 1A, SS chamber). As a control, a virgin male was similarly placed in a larger chamber where it could walk freely (Figure 1A, standard chamber). Hereafter, males exposed to SS stress are referred to as “stressed males”, whereas control males are referred to as “naive males”. Following the stress exposure, courtship tests were performed using both naive and stressed males. Male courtship behavior toward an immobilized female was observed in an observation chamber (15 mm diameter, 3 mm depth) immediately (0 h) after the SS stress experience. Male courtship activity was quantified using the courtship index (CI), defined as the percentage of time spent performing courtship behaviors during a 10 min period.

Males exposed to 10 min of SS stress showed no significant difference from naive males in CI (Figure 1B). Naive males confined for 10 min in the standard chamber displayed lower CIs than those confined in the standard chamber for 30 or 60 min (10 min vs. 30 min, Steel-Dwass test, *q* = 3.078, *p* = 0.0059; 10 min vs. 60 min, Steel-Dwass test, *q* = 3.1136, *p* = 0.0053), likely due to the residual effects of cold anesthesia administered before confinement. Unlike the short 10-min exposure, males subjected to 30 or 60 min of SS stress exhibited a marked reduction in CI compared with naive controls (Figure 1B), demonstrating that longer confinement durations effectively induce courtship suppression. Moreover, CI in males subjected to 60 min of SS stress was significantly lower than that in males subjected to 30 min of SS stress (Steel-Dwass test, *q* = 2.5105, *p* = 0.0323), indicating that the degree of inhibition increased with the duration of SS stress. This suggests a duration-dependent relationship between SS stress and courtship suppression. From these findings, a 1-h SS stress protocol was used in subsequent experiments to ensure consistent and robust induction of courtship suppression.

We next examined how long the suppression persisted after SS stress exposure. Courtship activity was measured immediately (0 h), 1, 2, and 4 h after 1-h SS stress (Figure 1C). When there was an interval of more than 1 h between confinement and tests, males were individually maintained in food vials (10 mm in diameter, 75 mm in height) after confinement. Stressed males showed significantly lower courtship activity immediately and 1 h after 1-h SS stress, but not at 2 or 4 h after SS stress (Figure 1C). However, no significant difference in the CI was detected between naive and stressed males 2 and 4 h after SS stress (Figure 1C). These results indicate that SS-stress-induced courtship suppression is transient and gradually diminishes after stress cessation. To determine whether the duration of the stress experience affects subsequent suppression, we measured courtship activity 1 h after 10-, 30-, or 60-min SS stress. Courtship suppression was evident only after 60 min of SS stress, but not after 30 or 10 min (Figure 1D), further confirming the duration dependence of this behavioral effect.

In the experiments described previously, the observation chamber used to assess male courtship behavior was identical in size to the standard chamber. Thus, it remained possible that reduced courtship activity in stressed males resulted from being transferred into a novel environment. To test this, we used a larger observation chamber (21 mm diameter, 3 mm depth) and compared males confined for 1 h in the SS (3 mm diameter), standard (15 mm), and large (21 mm) chambers. Courtship behavior was then recorded immediately or 1 h after confinement (Figure 1E). Only males confined in the SS chamber exhibited significant courtship suppression immediately and 1 h after SS stress (Figure 1E, gray boxes). These results indicate that courtship suppression following confinement in SS chambers is a stress-induced response caused by movement restriction rather than a reaction to environmental novelty.

To assess whether the courtship suppression following SS stress observed in the CS strain is also present in other wild-type strains with different genetic backgrounds, we tested two additional wild-type strains, Oregon-R (OR) and Q-140. Both strains displayed significant courtship suppression immediately and 1 h after 1-h SS stress (Figure 1F). Although naive Q-140 males had lower baseline CIs than CS males 1 h after SS stress (Steel-Dwass test: CS vs. Q-140, *q* = 3.35, *p* = 0.002), SS stress still produced a clear suppressive effect. These findings indicate that SS stress exposure reliably suppresses courtship activity regardless of genetic background or baseline mating drive.

Previous studies have shown that repeated vibration stress induces a low motivational state in *Drosophila*, reducing spontaneous locomotor activity, appetite, and sexual desire.^7^ To determine whether SS stress has broader behavioral effects, we measured spontaneous locomotor activity and feeding behavior. Locomotor activity was significantly reduced immediately after 1-h SS stress (Figure S1A, 0 h) but returned to the baseline 1 h later (Figure S1A, 1 h), indicating that courtship suppression observed 1 h after 1-h SS stress is not simply due to general sluggishness. Feeding behavior was next assessed using the automated feeding monitoring system, FlyPAD.^31^ After a 23-h fasting period, feeding activity (number and duration of sips) was monitored for 1 h after 1-h SS stress. No significant differences were detected between naive and stressed males (Figure S1B), indicating that SS stress has a minimal impact on male appetite.

Finally, we examined whether prolonged-confinement-induced stress extends the duration of subsequent courtship suppression. Male courtship activity was measured on day 1 (d 1) and day 5 (d 5) after 7-h or 24-h SS stress. Males exhibited markedly reduced courtship activity on the day after 7-h and 24-h SS stress (Figure S2A), without accompanying decreases in locomotor activity (Figure S2B). Notably, courtship suppression persisted for at least 5 days after confinement (Figure S2A), indicating that the duration of courtship suppression is strongly affected by the length of the stress experience.

### Dopamine synthesis is required to sustain SS-stress-induced suppression of male courtship

**Figure 2:**
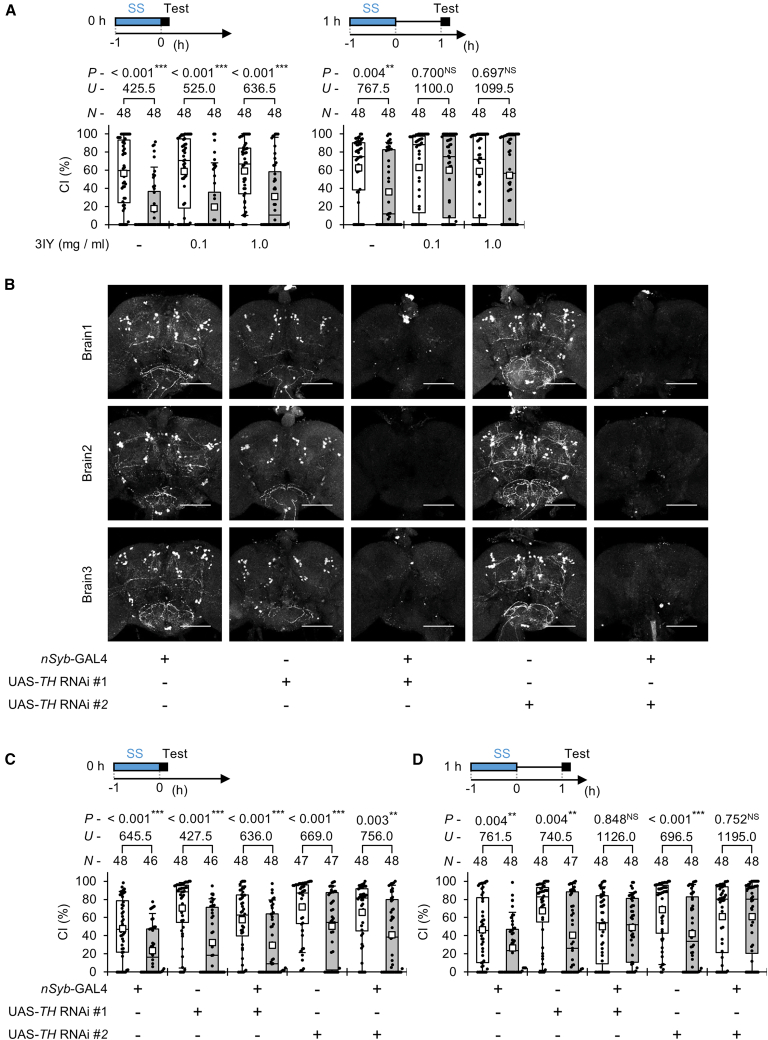
Dopamine is involved in the persistence of courtship suppression after SS stress experience (A) CS males with or without 3IY feeding were used in the experiments. Courtship activity was measured immediately (0 h) and 1 h after 1-h SS stress. 3IY feeding was conducted for 2 days before the experiments. (B) Antibody staining of the adult brains from *TH* knockdown males. Representative images of male brains stained with an anti-TH antibody are shown for each genotype. All confocal images of the adult brain were taken from the posterior side. Scale bars indicate 100 μm. Three brains were used for the experiments. (C and D) Male courtship activity was measured 1 h after 1-h SS stress using *TH* knockdown flies. *TH*-RNAi #1 or *TH*-RNAi #2 was driven by *nSyb*-GAL4. Courtship activity was measured immediately (C) and 1 h (D) after 1-h SS stress. (A, C, and D) We visualized the data using a boxplot with individual data points (black dots). In each graph, white boxes indicate naive males, and gray boxes indicate stressed males. Boxplots for a set of CI data show the 10th, 25th, 75th, and 90th centiles. In the boxplots, white squares indicate the mean, and the lines are drawn at the median. For statistical comparisons, the Mann-Whitney *U* test was used for CI. *n*, sample size; ∗∗∗, *p* < 0.001; ∗∗, *p* < 0.01; NS, not significant.

To examine the role of dopamine in SS-stress-induced courtship suppression, we used males fed with 3-iodo-L-tyrosine (3IY), an inhibitor of TH—the enzyme that catalyzes the conversion of L-tyrosine to L-DOPA, a precursor of dopamine.^32^ Feeding 3IY disrupts dopamine synthesis and has been shown to reduce dopamine levels in both *Drosophila* larvae and adults.^33^^,^^34^ In our experiments, 3-day-old virgin males were fed 3IY (0.1 or 1 mg/mL) for 2 days prior to the tests. Feeding 3IY did not affect courtship suppression immediately after SS stress (Figure 2A, left). However, 1 h after stress exposure, 3IY-fed males no longer exhibited courtship suppression (Figure 2A, right). These results suggest that dopamine synthesis is not required for the initial induction of courtship suppression but is necessary for maintaining the suppression after the stress experience.

To further confirm the involvement of dopamine synthesis, we performed pan-neuronal knockdown of TH using the *nSyb*-GAL4 driver and two UAS-*TH* RNAi lines (UAS-*TH* RNAi #1 [TRiP HMS05881] and UAS-*TH* RNAi #2 [TRiP HMC06137]). Immunostaining with an anti-TH antibody revealed that TH expression in the adult brain was almost completely abolished in *TH* knockdown flies (Figure 2B). Behaviorally, these flies (*nSyb*-GAL4/UAS-*TH* RNAi) showed normal courtship suppression immediately after SS stress (Figure 2C) but failed to maintain the suppression 1 h later (Figure 2D).

In naive males shown in Figure 2C, the CIs of *nSyb*-GAL4/UAS-*TH* RNAi #1 and *nSyb*-GAL4/UAS-*TH* RNAi #2 did not differ significantly from their respective UAS control males (Table S1), and similar results were observed for the data shown in Figure 2D (Table S2). These findings indicate that *TH* knockdown alone does not impair baseline male courtship activity. Together, these results demonstrate that dopamine synthesis in neurons is crucial for sustaining SS-stress-induced suppression of courtship behavior, while the initial induction of suppression immediately after SS stress occurs through a dopamine-independent mechanism.

### Dopamine release during and after SS stress is required to sustain courtship suppression

We next examined whether inhibiting dopamine release during or after SS stress affects stress-induced courtship suppression. To block dopamine release, we expressed the temperature-sensitive dynamin mutation s*hibire*^*ts1*^ (*shi*^*ts1*^) in dopamine neurons using *TH*-GAL4 and UAS- *shi*^*ts1*^ lines. Because the expression pattern of TH-GAL4 closely matches that of endogenous TH, *TH*-GAL4 serves as a reliable driver for dopamine neurons.^35^ Shi^ts1^ can inhibit synaptic vesicle recycling in a temperature-dependent manner, thereby reversibly blocking neurotransmission at restrictive temperatures.^36^

**Figure 3:**
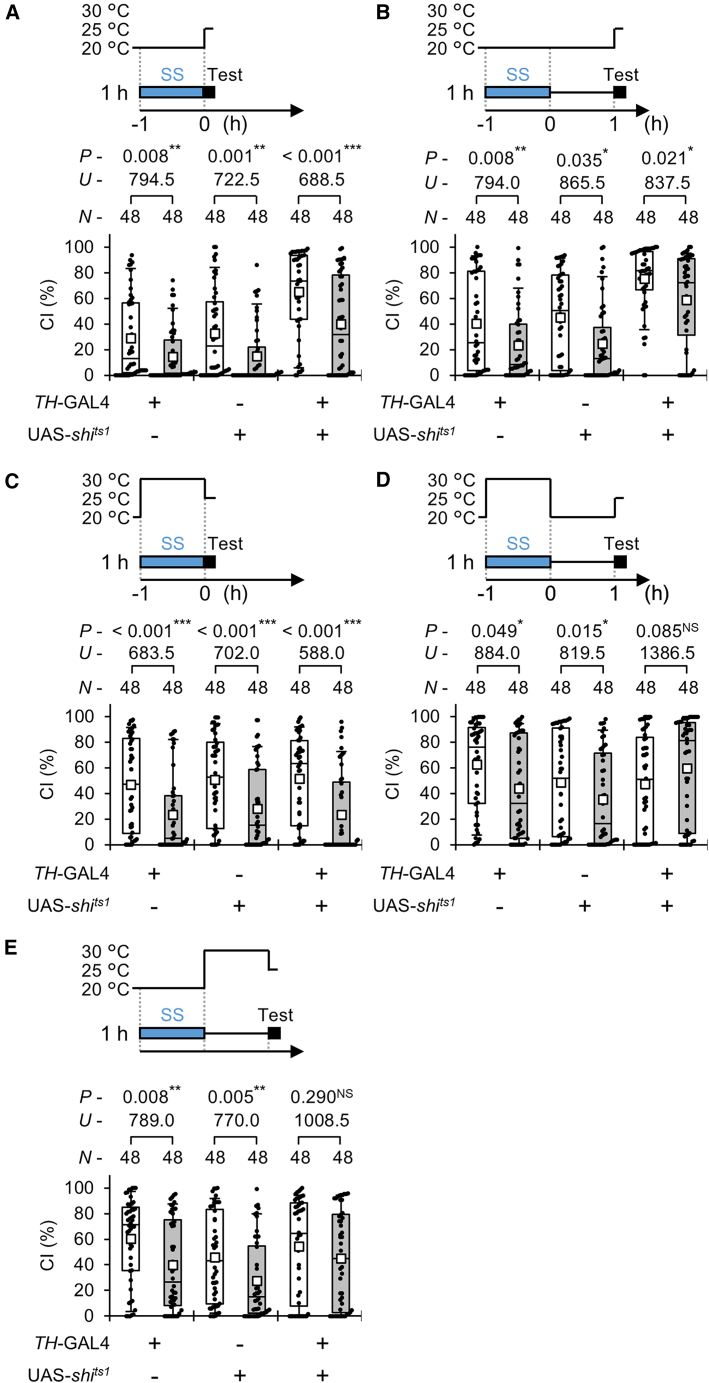
Disruption of neurotransmission from dopamine neurons during and after exposure to SS stress inhibits courtship suppression after the stress experience *shi*^*ts1*^ was driven by *TH*-GAL4. Virgin males were collected and kept at 20 °C until the experiments. (A) Male courtship activity was measured at 25 °C after 1-h SS stress at PT (20 °C). (B) After 1-h SS stress at PT (20 °C) followed by 1-h maintenance of stressed flies at 20 °C, male courtship activity was measured at 25°C. (C) Male courtship activity was measured at 25 °C after 1-h SS stress at RT (30 °C). (D) After 1-h SS stress at RT (30 °C) followed by 1-h maintenance of stressed flies at 20 °C, male courtship activity was measured at 25°C. (E) After 1-h SS stress at PT (20 °C) followed by 1-h maintenance of stressed flies at 30 °C, male courtship activity was measured at 25 °C. (A–E) We visualized the data using a boxplot with individual data points (black dots). In each graph, white boxes indicate naive males, and gray boxes indicate stressed males. Boxplots for a set of CI data show the 10th, 25th, 75th, and 90th centiles. In the boxplots, white squares indicate the mean, and the lines are drawn at the median. For statistical comparisons, the Mann-Whitney *U* test was used for CI. *n*, sample size; ∗∗∗, *p* < 0.001; ∗∗, *p* < 0.01; ∗, *p* < 0.05; NS, not significant.

First, we assessed male courtship behavior at a permissive temperature (PT) of 25 °C after exposure to SS stress for 1 h at a PT of 20 °C (Figures 3A and 3B). Both *TH*-GAL4/UAS-*shi*^*ts1*^ males and control males displayed significant courtship suppression immediately after SS stress (Figure 3A) and again 1 h later (Figure 3B). In naive males, the CI of *TH*-GAL4/UAS-*shi*^*ts1*^ flies was significantly higher than those of the GAL4 and UAS controls (Tables S3 and S4), whereas no significant differences were detected between the two control genotypes (Tables S3 and S4). These results suggest that *TH*-GAL4/UAS-*shi*^*ts1*^ males exhibit elevated baseline courtship activities under temperature shift conditions (20°C–25°C). However, this increased baseline activity did not alter their susceptibility to SS stress, as courtship suppression was still observed, similar to that in control males.

To determine whether dopamine release during the stress experience contributes to courtship suppression, we next subjected flies to 1 h of SS stress at the restrictive temperature (RT, 30 °C). Under this condition, *TH*-GAL4/UAS-*shi*^*ts1*^ males showed normal courtship suppression immediately after 1-h SS stress, comparable to control males (Figure 3C). This finding indicates that dopamine release during the stress period is not required for the immediate induction of courtship suppression. In contrast, *TH*-GAL4/UAS-*shi*^*ts1*^ males failed to exhibit courtship suppression 1 h after the SS stress (Figure 3D), whereas control males maintained reduced courtship suppression (Figure 3D). Next, flies were subjected to SS stress for 1 h at a PT of 25 °C and then kept at RT for 1 h before the tests at a PT of 25 °C. Under this condition, *TH*-GAL4/UAS-*shi*^*ts1*^ males again failed to show courtship suppression, whereas control males continued to do so (Figure 3E). Collectively, these results demonstrate that dopamine release during and after SS stress is essential for maintaining, but not initiating, stress-induced courtship suppression.

### Three types of dopamine receptor are responsible for the persistence of courtship suppression after stress experience

**Figure 4:**
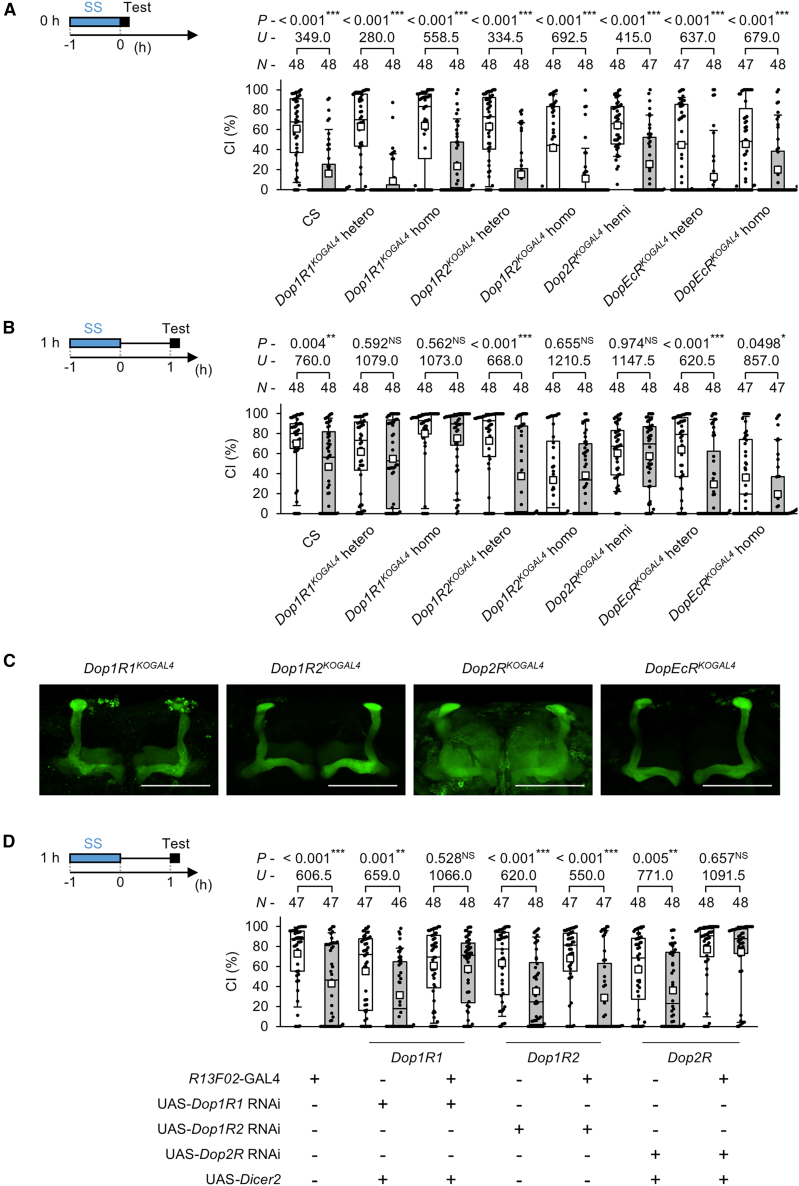
Dopamine receptors are responsible for the persistence of courtship suppression after SS stress experience (A) Courtship activity was measured immediately after 1-h SS stress. (B) Courtship activity was measured 1 h after 1-h SS stress. (A and B) knockout GAL4 lines of four dopamine receptor genes were used in the experiments (*Dop1R1*^*KOGAL4*^, *Dop1R2*^*KOGAL4*^, *Dop2R*^*KOGAL4*^, and *DopEcR*^*KOGAL4*^). (C) Stacked confocal images of the MB and its surrounding regions in the adult brain. We used F_1_ males between knockout GAL4 lines of four dopamine receptor genes and the UAS-*mCD8::GFP* line. Scale bars represent 100 μm. Green, mCD8::GFP. (D) Male courtship activity was measured 1 h after 1-h SS stress. Males with *Dop1R1*, *Dop1R2*, or *Dop2R* knockdown in the MB were used. (A, B, and D) We visualized the data using a boxplot with individual data points (black dots). In each graph, white boxes indicate naive males and gray boxes indicate stressed males. Boxplots for a set of CI data show the 10th, 25th, 75th, and 90th centiles. In the boxplots, white squares indicate the mean, and the lines are drawn at the median. In statistical analyses, the Mann-Whitney *U* test was used for CI. *n*, sample size; ∗∗∗, *p* < 0.001; ∗∗, *p* < 0.01; ∗, *p* < 0.05; NS, not significant. See also Figure S3.

*Drosophila* has four dopamine receptors, all of which are G-protein-coupled receptors: Dop1R1, Dop1R2, Dop2R, and DopEcR.^37^^,^^38^ Dop1R1 and Dop1R2 are D1-like receptors that activate the cAMP signaling pathway,^37^ whereas Dop2R is a D2-like receptor believed to inhibit the cAMP pathway.^37^ Additionally, flies have a noncanonical dopamine receptor, DopEcR, which is activated by both dopamine and ecdysone.^37^ To determine which dopamine receptors are required for male courtship suppression after SS stress, we used knockout (KO) GAL4 lines for each dopamine receptor.^39^ These KO GAL4 lines are null mutants, as one or more exons of each dopamine receptor gene are replaced by GAL4. First, we observed courtship behavior immediately after SS stress. Recording the CI of naive males, no significant differences were observed between CS and KO GAL4 lines (Figure 4A; Table S5). Furthermore, courtship suppression immediately after 1-h SS stress was detected in all four KO GAL4 lines (Figure 4A), indicating that the absence of any one of the dopamine receptors does not affect the immediate response. However, when examining the persistence of courtship suppression, we found that homozygotes or hemizygous males of three KO GAL4 males (*Dop1R1*^*KOGAL4*^, *Dop1R2*^*KOGAL4*^, and *Dop2R*^*KOGAL4*^) did not exhibit courtship suppression after 1-h SS stress (Figure 4B). In contrast, males lacking *DopEcR* (*DopEcR*^*KOGAL4*^) continued to show courtship suppression (Figure 4B), even though their naive male counterparts showed significantly lower CIs than CS controls (Table S6). Thus, DopEcR is unlikely to mediate SS-stress-induced courtship suppression. Similarly, naive males homozygous for *Dop1R2*^*KOGal4*^ also showed significantly lower CIs than CS controls (Table S6), and their CIs were comparable to those of *DopEcR*^*KOGal4*^ (*DopEcR*^*KOGal4*^ homo vs. *Dop1R2*^*KOGal4*^ homo, *U* = 1060.0, *p* = 0.599). However, males homozygous for *Dop1R2*^*KOGal4*^ did not show courtship suppression 1 h after SS stress (Figure 4B). Thus, Dop1R2 may be required to maintain stress-dependent courtship suppression.

### Dopamine receptors in MB neurons are involved in the persistence of courtship suppression after stress experience

In *Drosophila*, dopamine receptors are expressed in mushroom body (MB) neurons,^39^^,^^40^ which are required for various *Drosophila* behaviors.^41^ Using F_1_ males generated by crossing a UAS-*mCD8*::*GFP* line and KO GAL4 lines for the four dopamine receptors, we confirmed that dopamine receptors are expressed in the MB, as previously reported (Figure 4C). Because DopEcR was not involved in the persistence of stress-dependent courtship suppression (Figure 4B), we focused our subsequent experiments on Dop1R1, Dop1R2, and Dop2R. To examine whether dopamine receptors in MB neurons contribute to stress-induced courtship suppression, we performed RNAi experiments targeting these three dopamine receptors. We used the UAS-*Dop1R1* RNAi, UAS-*Dop1R2* RNAi, and UAS-*Dop2R* RNAi lines in combination with the pan-MB GAL4 line *R13F02*. The TRiP RNAi line UAS-*Dop1R2* RNAi, constructed using the VALIUM20 vector, is effective without the enforced expression of *Dicer2* (Bloomington Drosophila Stock Center: https://bdsc.indiana.edu/stocks/rnai/rnai_all.html); thus, this line was used independently of UAS-*Dicer2*. Conversely, the other UAS-RNAi lines were combined with UAS-*Dicer2* to enhance RNAi efficiency.

The knockdown effectiveness of all three UAS-RNAi lines was confirmed by quantitative reverse-transcription PCR (RT-qPCR) using the pan-neuronal *nSyb*-GAL4 line (Figure S3). Behavioral analysis revealed that males with *DopR1*or *Dop2R* knockdown did not show courtship suppression 1 h after 1-h SS stress, whereas males with *Dop1R2* knockdown retained this suppression (Figure 4D). In addition, knockdown of *Dop1R1*, *Dop1R2*, and *Dop2R* expression in the MB did not affect CI in naive males (Table S7). Taken together, Dop1R1 and Dop2R in MB neurons contribute to maintaining courtship suppression following SS stress.

### Neurotransmission in PAM and PPL1 neurons is involved in the persistence of courtship suppression after stress experience

**Figure 5:**
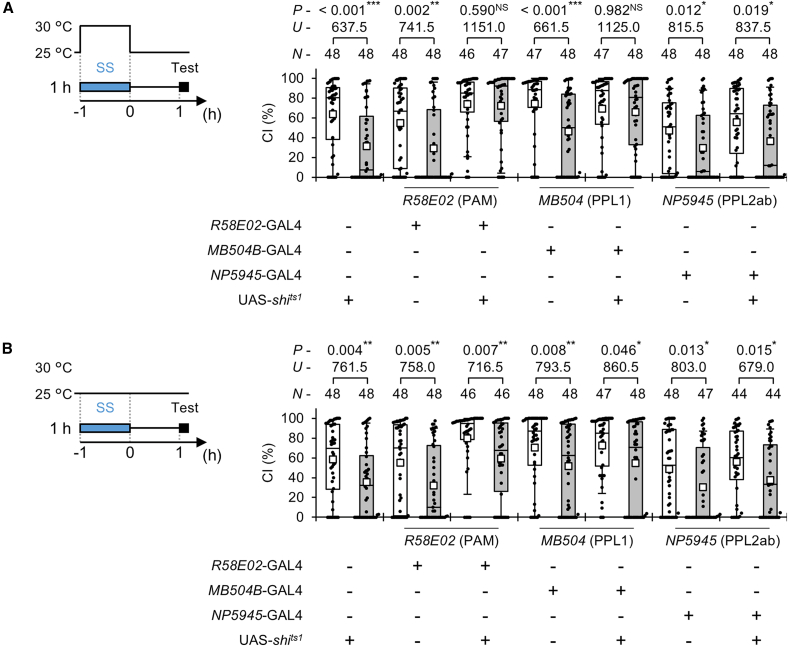
Disruption of neurotransmission in PAM and PPL1 prevents the persistence of courtship suppression after SS stress To induce *shi*^*ts1*^ expression in PAM, PPL1, and PPL2ab, three GAL4 lines were used (*R58E02* for PAM, *MB504B* for PPL1, and *NP5945* for PPL2ab). (A) SS stress was applied at 30 °C for 1 h followed by the maintenance of stressed flies at 25 °C for 1 h, and then courtship was observed at 25°C. (B) SS stress was applied at 25 °C for 1 h followed by the maintenance of stressed flies at 25 °C for 1 h, and then courtship was observed at 25 °C. (A and B) We visualized the data using a boxplot with individual data points (black dots). In each graph, white boxes indicate naive males and gray boxes indicate stressed males. Boxplots for a set of CI data show the 10th, 25th, 75th, and 90th centiles. In the boxplots, white squares indicate the mean, and the lines are drawn at the median. For statistical comparisons, the Mann-Whitney *U* test was used for CI. *n*, sample size; ∗∗∗, *p* < 0.001; ∗∗, *p* < 0.01; ∗, *p* < 0.05; NS, not significant.

In *Drosophila*, the adult brain contains approximately 300 dopamine neurons, which are classified into nine clusters (PAM, PAL, PPM1, PPM2, PPM3, PPL1, PPL2ab, PPL2c, and T1).^35^^,^^42^ Among these clusters, PAM, PPL1, and PPL2ab dopamine neurons terminate and innervate MB neurons.^41^ Thus, we next examined whether inhibiting neurotransmitter release from these clusters during SS stress exposure can prevent the persistence of courtship suppression after SS stress experience. We used two GAL4 lines (*R58E02* for PAM and *NP5945* for PPL2ab) and a split-GAL4 line (*MB504B* for PPL1). In *R58E02*/UAS-*shi*^*ts1*^ and *MB504B*/UAS-*shi*^*ts1*^ males, no courtship suppression was observed 1 h after 1-h SS stress when SS stress was applied at RT (30 °C) (Figure 5A), and disruption of neurotransmission in these three dopamine clusters did not affect naive CI at RT (Table S8). In contrast, suppression was present when the stress was conducted at PT (25 °C) (Figure 5B). The CI of naive *R58E02*/UAS-*shi*^*ts1*^ males was significantly higher than that of control males at PT (Table S9). However, courtship suppression following SS stress was still detected, as was observed in the two control genotypes (Figure 5B). This result indicates that increased baseline courtship activity did not alter their susceptibility to SS stress. Taken together, the results suggest that dopamine release from PAM and PPL1 neurons is critical for maintaining courtship suppression for at least 1 h after 1-h SS stress. Unlike *R58E02* and *MB504B* lines, *NP5945*/UAS-*shi*^*ts1*^ males showed courtship suppression at both PT and RT (Figures 5A and 5B), indicating that blocking dopamine release from PPL2ab has little effect on SS-stress-induced courtship suppression.

## Discussion

Stress experiences cause sexual dysfunctions in mammals,^19^^,^^21^^,^^43^ yet the molecular and cellular mechanisms underlying these effects remain largely unclear. In this study, we demonstrated that *Drosophila* males show courtship suppression following confinement in a small space without complete immobilization (SS stress). A 1-h SS stress exposure significantly reduced male courtship activity for at least 1 h (Figure 1), whereas longer SS stress exposures of 7 h and 24 h induced suppression lasting for at least 5 days (Figure S2). These findings indicate that the duration of stress exposure affects the persistence of stress-induced behavioral suppression. In *Drosophila*, other stressors such as starvation or heat shock do not strongly affect male courtship activity.^44^^,^^45^ However, Ries et al. reported that prolonged vibration stress (300 Hz vibration for 10 h per day) reduced motivation for behaviors related to sexual desire, locomotion, and feeding.^7^ In contrast, our study found that SS stress selectively impaired courtship behavior without affecting general locomotor activity or appetite (Figure S1). This suggests that different stress types, or differences in exposure duration, uniquely affect neuronal circuits governing specific behavior.

We analyzed male courtship behavior across several wild-type and transgenic lines. The baseline CIs of naive males varied among these genotypes; for instance, the CI of Q-140 males was significantly lower than that of CS (Figure 1). In contrast, *TH*-GAL4/UAS-*shi*^*ts1*^ males and *R58E02*/UAS-*shi*^*ts1*^ males exhibited a significantly higher CI than their parental GAL4 and UAS control males (Tables S3, S4, and S9), likely reflecting a synergistic effect of the two transgenes on courtship behavior, even though both had been backcrossed to a CS background. Importantly, however, courtship suppression after SS stress was observed consistently across all genotypes, indicating that confinement-induced stress reliably suppresses male courtship regardless of baseline courtship levels.

Using three independent approaches—temporal inhibition of TH using 3IY, RNAi-mediated *TH* knockdown, and blocking neurotransmission in dopamine neurons—we found that dopamine synthesis and neurotransmission are required for the persistence of courtship suppression following SS stress (Figures 2 and 3). In contrast, inhibiting dopamine synthesis or neurotransmission had little effect on the immediate suppression of courtship behavior after SS stress (Figures 2 and 3). These results indicate that dopamine signaling is not a major contributor to the initial phase of courtship suppression, and suggest that other neurotransmitters likely play a more prominent role in the immediate response. Furthermore, we found that synaptic transmission from PAM and PPL1 clusters to the MB (Figure 3), along with DopR1 and Dop2R expressed in the MB (Figure 4), is necessary to sustain courtship suppression following SS stress. These results indicate that the dopamine signaling pathway from PAM and PPL1 to MB is required for maintaining SS-stress-induced courtship suppression.

From these findings, we propose a model for the sustained courtship suppression induced by SS stress. In this model, SS stress activates non-dopaminergic pathways that initiate courtship suppression independently of dopamine neurons. At the same time, SS stress also activates dopamine neurons and enhances dopamine release. This enhanced dopamine signaling during stress modifies the neuronal circuits that regulate courtship motivation, such that the suppression of courtship behavior is subsequently maintained in a dopamine-dependent manner. Future electrophysiological and imaging studies will be required to elucidate the neuroplastic mechanisms underlying this sustained suppression of courtship behavior.

In *Drosophila*, repeated mating leads to a progressive reduction in male courtship motivation toward females.^30^ The male courtship command neurons, P1 neurons, express the dopamine receptor Dop1R2. As mating experience accumulates, dopaminergic input to P1 neurons decreases, resulting in reduced male courtship motivation. In contrast, our findings suggest that stress activates dopamine release from at least the PAM and PPL1 dopaminergic clusters, and that this stress-induced dopaminergic activation is also associated with reduced male courtship motivation. Together, these observations indicate that dopamine signaling plays a central role in regulating male courtship motivation. However, the effects of dopamine are not uniform; rather, the impact on courtship motivation likely depends on the specific dopaminergic neuron populations and circuits engaged. Thus, distinct dopaminergic pathways may exist that either promote or suppress male courtship motivation.

An experience-dependent form of behavioral plasticity in *Drosophila*, known as courtship conditioning, has been described, in which male courtship activity is suppressed after specific social experience.^46^ During courtship conditioning, males encounter aversive cues from mated females (e.g., courtship-inhibiting signals and sexual rejection) that prevent successful mating and subsequently decrease courtship activity even toward virgin females.^47^^,^^48^ A key question is whether courtship suppression induced by SS stress involves mechanisms similar to those underlying courtship conditioning. Courtship suppression induced by courtship conditioning is also regulated by dopamine signaling: dopamine release primarily from aSP13 neurons, a subset of the PAM cluster, and Dop1R1 activity in MB neurons are essential for this process.^49^ In contrast, our study indicates that SS stress-induced suppression involves dopamine release from both PAM and PPL1 clusters, as well as Dop1R1 and Dop2R signaling in MB neurons, contributing to persistent courtship suppression. Thus, although both paradigms engage dopaminergic modulation of MB circuits, SS stress-induced courtship suppression likely involves a broader and more complex dopaminergic mechanism than classical courtship conditioning.

In mammals, immobilization or restraint stress—often involving confinement in a small tube—affects various behaviors, including anxiety and depression-like behaviors.^50^^,^^51^^,^^52^ During immobilization, rodents experience strong psychological stress due to the inability to move freely, and this paradigm is widely used to model human mental disorders. Acute immobilization stress in mice suppresses escape-related behaviors during the tail suspension test, a process involving D3-type dopamine receptor signaling.^50^ Moreover, both acute and chronic immobilization stresses increase *TH* mRNA expression in the locus coeruleus of rats,^53^ supporting the idea that dopamine activity is altered by restraint stress. Our SS stress model shares conceptual similarities with mammalian stress paradigms with restricted free walking. Although *Drosophila* under SS stress can move their legs and body axis, the inability to walk freely likely induces psychological stress comparable to confinement or restraint in vertebrates. Thus, both models involve restricted movement and alterations in dopamine signaling. These parallels suggest that dopamine-mediated stress responses to restricted locomotion may represent a conserved phenomenon across vertebrates and invertebrates. Supporting this notion, restraint stress in male rats has been shown to induce sexual dysfunction, including reduced sexual motivation and erectile deficits.^54^^,^^55^ However, it remains unclear whether plastic changes in brain neurons via dopamine signaling underlie restraint-stress-induced sexual dysfunction, and this should be addressed in future studies.

The SS stress paradigm introduced in this study provides a model for investigating psychological stress in *Drosophila*. The stress likely arises from the inability to satisfy the innate drive for locomotion—a condition known to act as a significant psychological stressor in many animals.^56^ Although the neuronal and physiological mechanisms linking confinement to altered brain function remain poorly understood, our model offers a powerful approach to dissecting them genetically and behaviorally. Similarly, in humans, solitary confinement is known to cause severe psychological distress,^57^ yet the underlying neurobiological bases remain elusive. Our findings highlight the value of *Drosophila* as a tractable system for exploring how confinement and restricted movement affect brain function and behavior, offering insights potentially relevant to understanding stress-related disorders across species.

### Limitations of the study

Our findings suggest that dopamine signaling is not a major contributor to the initial phase of courtship suppression. However, the mechanisms underlying the immediate reduction in courtship activity remain elusive. Identifying the neurotransmitters that trigger the decrease in courtship activity during SS stress will be crucial for elucidating the initial regulatory pathways of SS-stress-induced courtship suppression.

As a next step, direct measurements of activity in dopamine-responsive neurons will be required to determine how their function is altered during and after stress. The adult *Drosophila* brain contains approximately 300 dopamine neurons organized into at least nine distinct clusters.^35^^,^^58^ Future studies should aim to determine which dopamine neuron clusters are activated during SS stress, and whether stress induces lasting functional changes in the downstream dopamine-responsive neurons. Neuronal activity indicators, such as GCaMP, allow real-time monitoring of neuronal activity in *Drosophila* brains.^59^ Although this approach is typically performed with the fly’s head fixed, locomotor restriction itself can affect dopamine neuron activity. Therefore, it will be important to assess dopamine neuron activity under both naturalistic locomotor conditions (e.g., using a trackball) and restricted conditions^60^ to fully understand how movement constraints modulate neuronal responses to stress.

After identifying dopamine neurons activated by SS stress, the next challenge will be to determine which downstream neurons receiving dopamine input are responsible for mediating courtship suppression. This study demonstrated that dopamine-responsive neurons in the MB contribute to this process. However, it remains elusive whether dopamine-responsive neurons outside the MB also play a role. Future work should clarify this point, as well as identify which dopamine receptor subtypes are expressed on these downstream neurons and how receptor function is altered following SS stress. These advances will be critical for understanding the molecular and neuronal mechanisms underlying stress-dependent sexual dysfunction.

## Resource availability

### Lead contact

Further information and requests for resources and reagents should be directed to and will be fulfilled by the lead contact, Takaomi Sakai (sakai-takaomi@tmu.ac.jp).

### Materials availability

No reagents were generated as part of this study.

### Data and code availability

• All data reported in this paper will be shared by the lead contact upon request.
• This paper does not report original code.
• Any additional information required to reanalyze the data reported in this paper is available from the lead contact upon request.

## Acknowledgments

This study was supported by 10.13039/501100001691JSPS 10.13039/501100001691KAKENHI (grant no. 21H02528 to T. Sakai) and a Grant-in-Aid for Scientific Research on Innovative Areas (Singularity Biology) from the 10.13039/501100001700Ministry of Education, Culture, Sports, Science and Technology of Japan (grant no. 21H00434 to T. Sakai). We thank Kahori Sasaki and Emiko Nakagawa for their technical assistance and Kohei Ueno for carefully reading the manuscript and providing critical comments. We also thank Shoma Sato and Show Inami for their helpful discussions. We are grateful to the Bloomington *Drosophila* Stock Center for providing the fly strains.

## Author contributions

Conceptualization, T. Sato and T. Sakai; formal analysis, T. Sato and R.T.; funding acquisition, T. Sakai; investigation, T. Sato and R.T.; methodology, T. Sato and T. Sakai; project administration, T. Sakai; supervision, T. Sakai and T.K.; validation, T. Sato; visualization, T. Sato, T.K., T. Sakai; writing – original draft, T.K. and T. Sakai; writing – review and editing, T.K. and T. Sakai.

## Declaration of interests

Authors declare no competing interests.

## STAR★Methods

### Key resources table

**Table:** 

REAGENT or RESOURCE	SOURCE	IDENTIFIER
**Antibodies**		

mouse anti-TH antibody	ImmunoStar	Cat#22941; RRID:AB_572268
goat anti-mouse IgG(H+L), Alexa Fluor 568	Thermo Fisher Scientific	Cat#A-11004; RRID:AB_2534072

**Chemicals, peptides, and recombinant proteins**		

3IY	Tokyo Chemical Industry	Cat#I0075
TRIzol Reagent	Thermo Fisher Scientific	Cat#15596018

**Critical commercial assays**		

PrimScript RT reagent Kit with gDNA Eraser (Perfect Real Time)	Takara Bio	Cat#RR047A
THUNDERBIRD SYBR qPCR Mix	TOYOBO	Cat#QPS-201

**Experimental models: organisms/strains**		

*D. melanogaster*: Canton-S (CS)	laboratory stock	N/A
*D. melanogaster*: Oregon-R (OR)	Tokyo Metropolitan University *Drosophila* collection	N/A
*D. melanogaster*: Q-140	Tokyo Metropolitan University *Drosophila* collection	N/A
*D. melanogaster*: *w*^*1118*^	Inami et al.^61^	N/A
*D. melanogaster*: *nSyb*-GAL4	Bloomington Drosophila Stock Center	RRID:BDSC_51635
*D. melanogaster*: *TH*-GAL4	Bloomington Drosophila Stock Center	RRID:BDSC_8848
*D. melanogaster*: *R58E02*-GAL4	Bloomington Drosophila Stock Center	RRID:BDSC_41347
*D. melanogaster*: *MB504B*-GAL4	gifted from Dr. Nakago, Tokyo Metropolitan Institute of Medical Science	RRID:BDSC_68329
*D. melanogaster*: *NP5945*-GAL4	Kyoto Drosophila Stock Center	RRID:DGGR_105062
*D. melanogaster*: *R13F02*-GAL4	Bloomington Drosophila Stock Center	RRID:BDSC_48571
*D. melanogaster*: *Dop1R1*^*KOGal4*^	Bloomington Drosophila Stock Center	RRID:BDSC_84714
*D. melanogaster*: *Dop1R2*^*KOGal4*^	Bloomington Drosophila Stock Center	RRID:BDSC_84715
*D. melanogaster*: *Dop2R*^*KOGal4*^	Bloomington Drosophila Stock Center	RRID:BDSC_84716
*D. melanogaster*: *DopEcR*^*KOGal4*^	Bloomington Drosophila Stock Center	RRID:BDSC_84717
*D. melanogaster*: UAS-*TH* RNAi #1	Bloomington Drosophila Stock Center	RRID:BDSC_76069
*D. melanogaster*: UAS-*TH* RNAi #2	Bloomington Drosophila Stock Center	RRID:BDSC_65875
*D. melanogaster*: UAS-*shi*^*ts1*^	Kitamoto^36^	N/A
*D. melanogaster*: UAS-*Dop1R1* RNAi	Bloomington Drosophila Stock Center	RRID:BDSC_93708
*D. melanogaster*: UAS-*Dop1R2* RNAi	Bloomington Drosophila Stock Center	RRID:BDSC_51423
*D. melanogaster*: UAS-*Dop2R* RNAi	Bloomington Drosophila Stock Center	RRID:BDSC_93711
*D. melanogaster*: UAS-*Dicer2*	Bloomington Drosophila Stock Center	RRID:BDSC_24650
*D. melanogaster*: UAS-*mCD8::GFP*	N/A	N/A

**Oligonucleotides**		

Primer: GAL4 Forward: AAAGAAAAACCGAAGTGCGCC	This paper	N/A
Primer: GAL4 Reverse: GGTCCGTTTTCAGGAAGGGC	This paper	N/A
Primer: *Dop1R1* Forward: TAGCGATTGCGGATCTCTTCG	This paper	N/A
Primer: *Dop1R1* Reverse: TGACATCAAAGGCCACCCAAG	This paper	N/A
Primer: *Dop1R2* Forward: TCGATAGAGAGAGCGAGTAGAGG	This paper	N/A
Primer: *Dop1R2* Reverse: TGATTCTGTTCCTGTTCCAATTTCC	This paper	N/A
Primer: *Dop2R* Forward: TCGCTGAGCAGCTTCTACATAC	This paper	N/A
Primer: *Dop2R* Reverse: CGTGAGTTCCGATAGGTGGG	This paper	N/A
Primer: *rp49* Forward: AAGATCGTGAAGAAGCGCAC	This paper	N/A
Primer: *rp49* Reverse: TGTGCACCAGGAACTTCTTG	This paper	N/A

**Software and algorithms**		

MATLAB (2019a)	MathWorks	RRID:SCR_001622
Move-tr/2D tracking software	Library	https://www.library-inc.co.jp/product/?id=1372146225-105767&ca=1
IBM SPSS Statistics (version 26 and 28)	IBM	RRID:SCR_016479
Excel Statistics (version 4.08)	Survey Research Information	RRID:SCR_017294

**Other**		

Nikon C2 confocal microscope	Nikon	N/A
Nikon AX confocal microscope	Nikon	N/A
LightCycler 96	Roche	Cat#05815916001

### Experimental model and study participant details

#### Fly stocks

All flies were raised on glucose–yeast–cornmeal medium in 12:12 LD cycles at 25.0 ± 0.5 °C (60 ± 20% relative humidity). In the behavioral experiments, virgin males and females were collected within 8 h after eclosion without anesthesia. Each virgin male was isolated until the experiments, except for the feeding assay. In all behavioral experiments, 3- to 6-day-old virgin males and wild-type virgin females were used. The analyses in this study focused primarily on male courtship behavior. The fly stocks of *D*. *melanogaster* used for this study were as follows: CS, OR, Q-140, *nSyb*-GAL4 [51635, Bloomington *Drosophila* Stock Center (BDSC)], *TH*-GAL4 (8848, BDSC), *R58E02* (41347, BDSC), *MB504B* (gifted from Dr. Nagano, Tokyo Metropolitan Institute of Medical Science), *NP5945* (105062, Kyoto *Drosophila* Stock Center), *R13F02* (48571, BDSC), *Dop1R1*^*KOGal4*^ (84714, BDSC), *Dop1R2*^*KOGal4*^ (84715, BDSC), *Dop2R*^*KOGal4*^ (84716, BDSC), *DopEcR*^*KOGal4*^ (84717, BDSC), UAS-*TH* RNAi #1 (76069, BDSC), UAS-*TH* RNAi #2 (65875, BDSC), UAS-*shi*^*ts1*^,^36^ UAS-*Dop1R1* RNAi (93708, BDSC), UAS-*Dop1R2* RNAi (51423, BDSC), UAS-*Dop2R* RNAi (93711, BDSC), UAS-*Dicer2* (24650, BDSC), and UAS-*mCD8::GFP*. Q-140 is a wild-type strain of *D. melanogaster* from the Tokyo Metropolitan University *Drosophila* collection, originally collected in Manila, Philippines, in 1979. All lines of transgenic flies were backcrossed to *white*^*1118*^ flies with the CS background for at least six generations except for *DopEcR*^*KOGal4*^, UAS-*mCD8::GFP*, UAS-*TH* RNAi #1, UAS-*TH* RNAi #2, and UAS-*Dop1R2* RNAi. *DopEcR*^*KOGal4*^ was backcrossed to CS flies for six generations. For backcrossing of *DopEcR*^*KOGal4*^, GAL4 insertion was confirmed in each generation by PCR analysis. Primer sequences used in the backcrossing are as follows: Forward, 5′-AAAGAAAAACCGAAGTGCGCC-3’; Reverse, 5′-GGTCCGTTTTCAGGAAGGGC-3’. The TRiP RNAi lines generated using the VALIUM20 vector used in this study (UAS-*TH* RNAi #1, UAS-*TH* RNAi #2, and UAS-*Dop1R2* RNAi) are expected to be effective even without being combined with UAS-*Dicer2* (https://bdsc.indiana.edu/stocks/rnai/rnai_all.html), so they were used without being combined with UAS-*Dicer2*.

### Method details

#### SS stress assay

3- to 6-day-old virgin males were used in the experiments. All experiments were conducted at 25 ± 0.5 °C (60 ± 20% relative humidity) except for the experiments using UAS-*shi*^*ts1*^. For cold anesthesia, males were collected into a chilled glass vial on ice within 5 min. Subsequently, each male was transferred into each well of the standard chambers (15 mm diameter, 3 mm depth, Figure 1A, right pictures), SS chambers (3 mm diameter, 2 mm depth, Figure 1A, left pictures), or large chambers (21 mm diameter, 3 mm depth). For 7-h and 24-h SS stress, males were fed fly food in the chambers to avoid desiccation and hunger. Except for those used in immediate tests, males were kept in small rearing vials with food (10 mm diameter, 75 mm height) until the tests after SS manipulation.

#### Behavioral analyses

In all experiments, 3- to 6-ayd-old wild-type virgin males were used. Tests of male courtship activity were carried out as previously described.^61^ Freeze-killed 3- to 6-day-old wild-type virgin females were used as tester females. In *D. melanogaster*, immobilized females have been used as tester females in studies of experience-dependent courtship suppression, such as courtship conditioning.^47^ Siegel and Hall, who first established the courtship conditioning, explained their use of immobilized females as tester females as follows: although active virgins are generally courted more persistently, the variation among trials is greater.^46^ Therefore, we also used immobilized females as tester females to measure courtship activity after stress experience. *D. melanogaster* wild-type males actively court females that have been freeze-killed immediately before the experiment.^62^ One male and one female as a couple were transferred into one well of an observation chamber (15 mm diameter, 3 mm depth). Then, courtship behaviors were videotaped for 10 min. CI was calculated as CI (%) = courting time (s)/600 (s) × 100.

In measuring spontaneous locomotor activity, a male was placed into one well of an observation chamber. Spontaneous locomotion was videotaped for more than 10 min. To reduce variability, the background was subtracted using a customized script of MATLAB (2019a, MathWorks, USA). The total travel distance from 5 to 605 s was measured using tracking software (Move-Tr/2D, Library, Japan).

In measuring feeding behavior, 20 to 30 virgin males were kept in separate vials until the experiment. For starvation induction, males were transferred to vials with 1% agarose gel 23 h before the feeding assay. After SS stress, the 1 h feeding behavior of males was monitored using FlyPAD.^31^ Electrodes on both sides in each arena were filled with 1% agarose gel with 10% sucrose as a food source. As indices of the appetite of flies, the number of sips and sip duration were used in this study. The numbers of sips detected in two electrodes were summed to obtain the total number of sips. For the sip duration, the measured sip durations in two electrodes were averaged. In all experiments using the GAL4/UAS system, F_1_ males obtained from crosses between the GAL4 line and wild-type CS and between the UAS line and CS were used as controls.

#### 3IY feeding

To inhibit *TH* in adults, we used standard fly food supplemented with 3IY (0.1 or 1.0 mg/ml) (I0075, Tokyo Chemical Industry, Japan). Flies were not starved prior to treatment; instead, they were maintained on 3IY-containing food for up to two days before the start of the experiment. After 1-h SS stress, control and 3IY-fed males were kept in breeding vials containing food except for those used in immediate tests.

#### Temporal disruption of neurotransmission by Shi^ts1^

*shi*^*ts1*^ was driven by four GAL4 lines (*TH*-GAL4, *R58E02*, *MB504B*, and *NP5945*). In this study, the RT and PT were 30 °C and 25 °C, respectively, except in the experiment using *TH*-GAL4. In the experiment using *TH*-GAL4, genetic controls and F_1_ hybrids were reared and used in experiments at 20 °C to minimize the effects of Shi^ts1^ at PT. The temperature of all chambers was pre-adjusted to RT and PT. All courtship measurements were conducted at 25 °C.

#### Immunohistochemistry

The fly brains were dissected in ice-chilled PBS. The dissected brain samples were fixed with 4% formaldehyde for 20 min at room temperature. The fixed brains were washed three times for 20 min with 0.2% Triton-X 100 and incubated with 1% normal goat serum (NGS) for 1 h. Primary antibody staining was conducted with a mouse anti-TH antibody (1:200) (22941, ImmunoStar, USA) at 4 °C for 2 d. Then, the samples were washed three times for 20 min with 0.2% Triton-X 100 and blocked for 30 min with 1% NGS. Secondary antibody staining was conducted with an anti-mouse IgG conjugated to Alexa Fluor 568 (1:1000) (A-11004, Thermo Fisher Scientific, USA) at 4 °C for 2 d. After washing three times for 20 min with 0.2% Triton-X 100, the brain samples were mounted with phosphate-buffered saline (PBS) and observed under a confocal microscope (C2, Nikon, Japan). Fluorescence was excited by a 561-nm laser, and images were obtained with a 20× objective lens (Plan Apo VC 20× DIC N2, Nikon, Japan). The pinhole size was 20.0 μm, and the z-interval was 0.85 μm. Samples from all genetic controls and F_1_ hybrids between the *nSyb*-GAL4 and UAS-*TH* RNAi lines were simultaneously processed using the same solutions for the validation of the presence of TH-KD.

#### Visualization of GAL4-expressing neurons

Fly brains were dissected in ice-chilled PBS. UAS-*mCD8::GFP* was driven by four knockout (KO) GAL4 lines (*Dop1R1*^*KOGal4*^, *Dop1R2*^*KOGal4*^, *Dop2R*^*KOGal4*^, and *DopEcR*^*KOGal4*^). Immediately after dissection, the brains were mounted in PBS and imaged using a confocal microscope (AX, Nikon, Japan). Fluorescence was excited with a 488-nm laser, and images were acquired with a 20× objective lens (Plan Apo VC 20× DIC N2, Nikon, Japan). The pinhole size was 16.1 μm, and the z-stack images were collected at 0.512 μm intervals.

#### Quantitative reverse transcription PCR analyses

Real-time quantitative reverse transcription PCR (qRT-PCR) analyses were carried out as previously described with some modifications.^63^ Total RNA was extracted from 30–40 heads of 3–8-day-old males using TRIzol (15596018, Thermo Fisher Scientific, USA) as one sample (210–320 heads were used for each genotype). cDNA was synthesized using an RT reagent kit with gDNA Eraser (RR047A, Takara Bio, Japan). qRT-PCR was carried out using THUNDERBIRD SYBR qPCR Mix (QPS-201, TOYOBO, Japan) and LightCycler 96 (05815916001, Roche, Swiss). The mRNA expression level of a target gene in each sample was measured as a ratio to the *rp49* mRNA expression level for internal control. Then, the ratio of mRNA expression levels of the target gene to that of *rp49* in each genotype was divided by that in *nSyb*-GAL4/+ flies for normalization. The following primers were used in the experiments: *Dop1R1*-Forward, 5′- TAGCGATTGCGGATCTCTTCG-3′; *Dop1R1*-Reverse, 5′-TGACATCAAAGGCCACCCAAG-3′; *Dop1R2*-Forward, 5′-TCGATAGAGAGAGCGAGTAGAGG-3′; *Dop1R2*-Reverse, 5′-TGATTCTGTTCCTGTTCCAATTTCC-3′; *Dop2R*-Forward, 5′-TCGCTGAGCAGCTTCTACATAC-3′; *Dop2R*-Reverse, 5′-CGTGAGTTCCGATAGGTGGG-3′; *rp49*-Forward, 5′-AAGATCGTGAAGAAGCGCAC-3′; *rp49-*Reverse, 5′-TGTGCACCAGGAACTTCTTG-3′.

### Quantification and statistical analysis

The sample number for each experiment is shown either in a figure or in the figure legend. All processed data supporting the findings are provided within the text. In all figures, asterisks indicate statistical significance: ∗∗∗, *P* < 0.001; ∗∗, *P* < 0.01; ∗, *P* < 0.05; NS, not significant.

All the statistical analyses were performed using IBM SPSS Statistics (version 26 and 28, IBM, USA) except for the Kruskal–Wallis test followed by the Steel–Dwass test. The Kruskal–Wallis test followed by the Steel–Dwass test was performed using BellCurve for Excel (version 4.08, Social Survey Research Information, Japan). All comparisons were performed as a two-sided test. In all statistical analyses, the Kolmogorov–Smirnov test and Levene’s test were used to test the normality and homoscedasticity, respectively. Non-normal distributions were mechanically transformed according to logarithmic, exponential, and square root transformations, and then normality was retested. In the statistical analysis of CI, all distributions did not show coherent normality even after the transformations. Thus, all data were analyzed using the nonparametric statistical tests. For comparisons between two groups, we applied the Mann–Whitney *U* test. For comparisons involving three or more groups, we performed nonparametric ANOVA (Kruskal–Wallis test) followed by the Steel–Dwass test.

In the statistical analysis of spontaneous locomotor activity, the Mann–Whitney *U* test was used for travel distances immediately after 1-h SS stress and 1 d after 24-h SS stress, and Student’s *t* test following logarithmic transformation was used for travel distances 1 h after 1-h SS stress and 1 d after 7-h SS stress. In the statistical analysis of feeding activity, the number of sips and sip duration were processed by Student’s *t* test as distributions showing normality and homoscedasticity.

In the statistical analysis of qRT-PCR results, all distributions showed normality. In *Dop1R1* and *Dop1R2* KD, distributions did not show coherent homoscedasticity. Thus, data were processed using the Games–Howell test following Welch’s ANOVA. In *Dop2R* KD, distributions showed homoscedasticity. Thus, data were processed using the Tukey HSD test following one-way ANOVA.
