# Supplementary material for: Role of dopamine signaling in male courtship suppression induced by confinement stress in Drosophila
Source: iScience. 2026 Apr 27;29(6):115906. doi: 10.1016/j.isci.2026.115906 (PMC13196396; doi:10.1016/j.isci.2026.115906)
Supplement: Document S1. Figures S1–S3 and Tables S1–S9 [file mmc1.pdf]

iScience, Volume *29*

Supplemental information

Role of dopamine signaling in male courtship suppression induced by confinement stress in *Drosophila*

Tomohito Sato, Rana Toyama, Toshihiro Kitamoto, and Takaomi Sakai

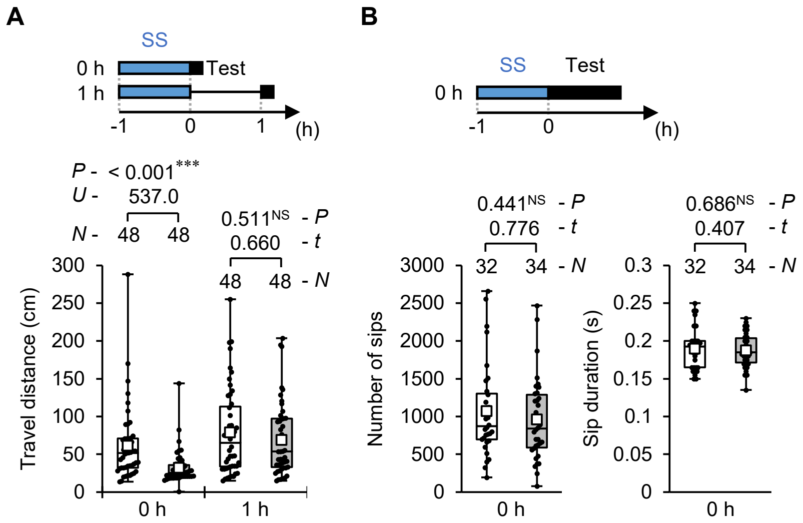

Figure S1. Spontaneous locomotor activity and feeding behavior after 1 h SS stress, Related to Figure1. (**A**) To measure general locomotor activity, the total travel distances for 10 min were measured immediately and 1 h after 1 h SS stress. (**B**) Feeding behavior was observed for 1 h after 1 h SS stress. The number of sips and sip duration in each male were measured using FlyPAD. (**A** and **B**) Wild-type (CS) males were used in the experiments. We visualized the data using a box plot with individual data points (black dots). In each graph, white boxes indicate naive males, and gray boxes indicate stressed males. Box plots show 0th, 25th, 75th, and 100th centiles. In the box plots, white squares indicate the mean, and the lines are drawn at the median. For statistical comparisons, the Mann–Whitney *U* test was used for the travel distance immediately after 1 h SS stress. Student's *t* test was used for the travel distances 1 h after 1 h SS stress and the number of sips and sip duration. *N*, sample size; ***, *P* < 0.001; NS, not significant.

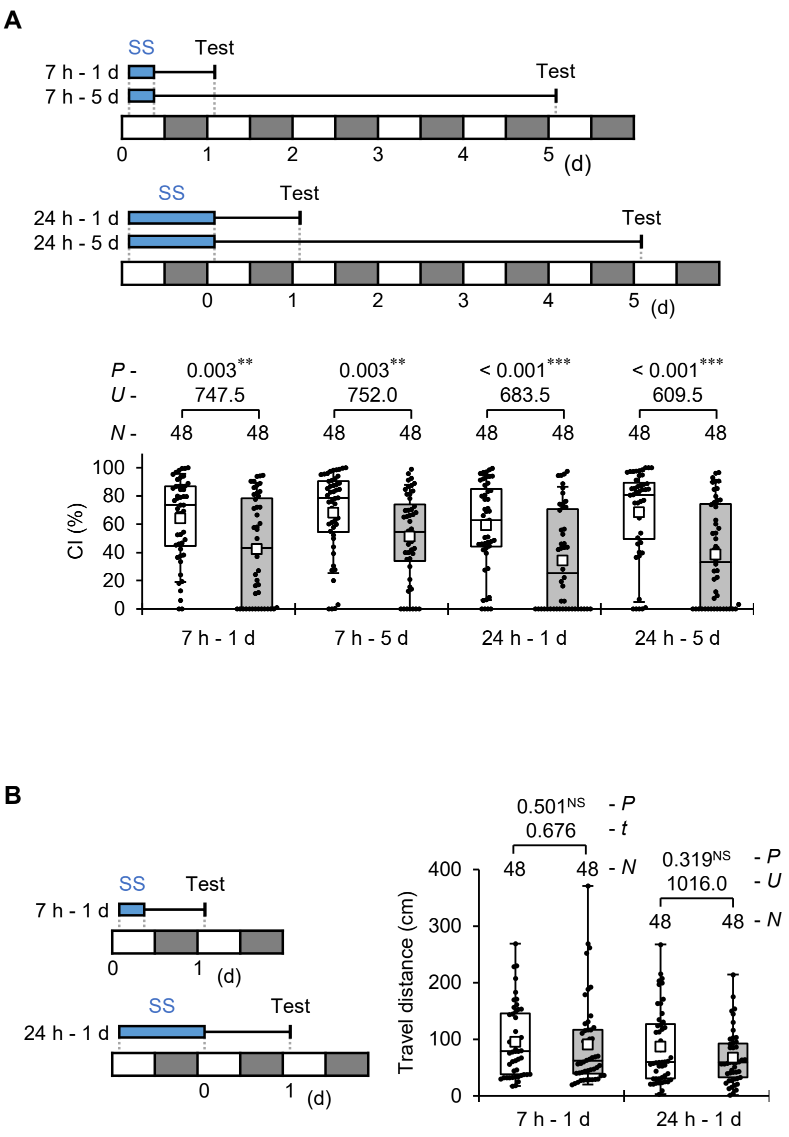

Figure S2. 7 h and 24 h SS-stress-induced courtship suppression lasts at least 5 d, Related to Figure1. (**A**) Courtship activity was measured 1 or 5 d after 7 or 24 h SS stress. (**B**) Total travel distances during 10 min spontaneous locomotor were measured 1 d after 7 or 24 h SS stress. (**A** and **B**) Wild-type (CS) males were used in the experiments. We visualized the data using a box plot with individual data points (black dots). In each graph, white boxes indicate naive males, and gray boxes indicate stressed males. Box plots for a set of CI data show the 10th, 25th, 75th, and 90th centiles, and those for a set of travel distance data show the 0th, 25th, 50th, 75th, and 100th centiles. In the box plots, white squares indicate the mean, and the lines are drawn at the median. For statistical comparisons, the Mann–Whitney *U* test was used for CI and travel distance 1 d after 7 h SS stress. Student's *t* test was used for travel distance 1 d after 7 h SS stress. *N*, sample size; ***, $P < 0.001$; **, $P < 0.01$; NS, not significant.

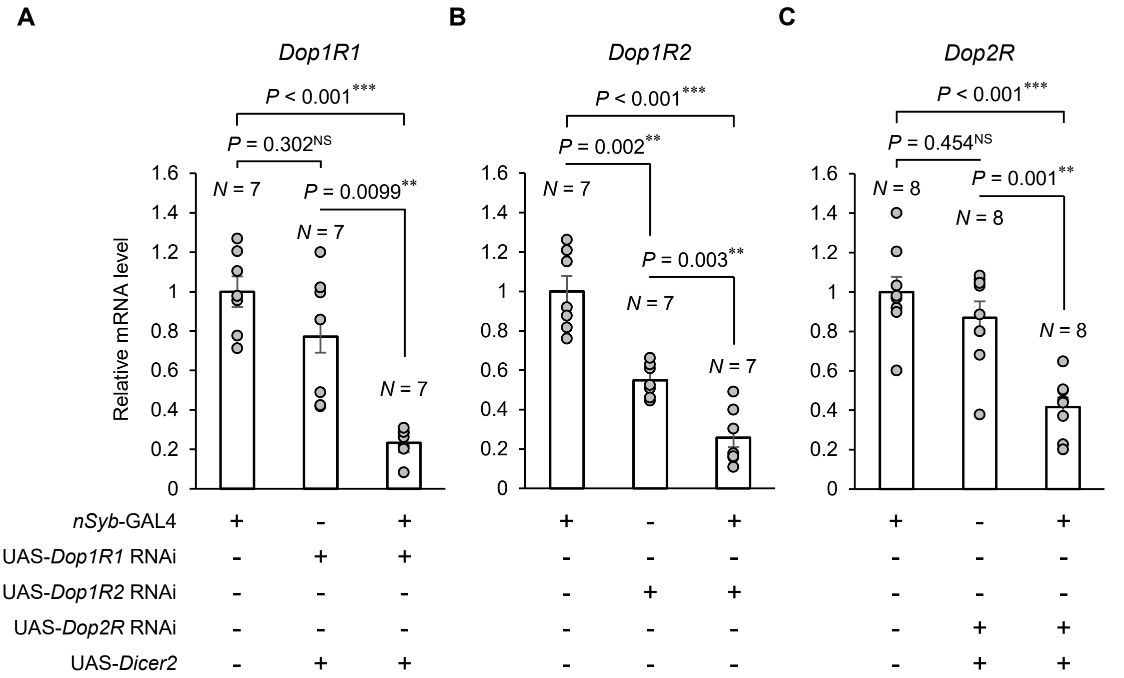

Figure S3. Real-time qRT-PCR analysis of *Dop1R1*, *Dop1R2,* and *Dop2R* knockdown, Related to Figure4D. The relative mRNA expression levels of *Dop1R1* (**A**), *Dop1R2* (**B**), and *Dop2R* (**C**) in each genotype were measured. (**A–C**) Total RNA was extracted from 30–40 male heads as one sample (210–320 heads were used for each group). Bar plots show the average ± SEM of each distribution with individual data points (gray circles). For statistical comparisons, the Games–Howell test following Welch's ANOVA was used for the comparison of relative *Dop1R1* and *Dop1R2* mRNA expression levels, and the Tukey HSD test following one-way ANOVA was used for the comparison of relative *Dop2R* mRNA expression levels. Results of ANOVA were as follows: *Dop1R1*, $F_{(2, 9.330)} = 44.676$, $P < 0.001$; *Dop1R2*, $F_{(2, 10.707)} = 29.688$, $P < 0.001$; *Dop2R*, $F_{(2, 21)} = 16.477$, $P < 0.001$. *N*, sample size; ***, $P < 0.001$; **, $P < 0.01$; NS, not significant.

Table S1. Statistical comparison between genotypes in the CI of naive males, related to Figure 2C

Genotypes	Steel–Dwass test (q)	P
nSyb-GAL4/+ vs UAS-*TH* RNAi #1/+	3.7885	0.0014
nSyb-GAL4/UAS-*TH* RNAi #1 vs *nSyb*-GAL4/+	1.6251	0.4812
nSyb-GAL4/UAS-*TH* RNAi #1 vs UAS-*TH* RNAi #1/+	2.2764	0.1525
nSyb-GAL4/+ vs UAS-*TH* RNAi #2/+	3.9465	< 0.001
nSyb-GAL4/UAS-*TH* RNAi #2 vs *nSyb*-GAL4/+	2.6934	0.0549
nSyb-GAL4/UAS-*TH* RNAi #2 vs UAS-*TH* RNAi #2/+	1.7421	0.4080

Table S2. Statistical comparison between genotypes in the CI of naive males, related to Figure 2D

Genotypes	Steel–Dwass test (q)	P
nSyb-GAL4/+ vs UAS-*TH* RNAi #1/+	2.5864	0.0728
nSyb-GAL4/UAS-*TH* RNAi #1 vs *nSyb*-GAL4/+	0.2981	0.9983
nSyb-GAL4/UAS-*TH* RNAi #1 vs UAS-*TH* RNAi #1/+	2.5941	0.0714
nSyb-GAL4/+ vs UAS-*TH* RNAi #2/+	2.7972	0.0412
nSyb-GAL4/UAS-*TH* RNAi #2 vs *nSyb*-GAL4/+	1.6019	0.4961
nSyb-GAL4/UAS-*TH* RNAi #2 vs UAS-*TH* RNAi #2/+	1.3457	0.6625

Table S3. Statistical comparison between genotypes in the CI of naive males, related to Figure 3A

Genotypes	Steel–Dwass test (q)	P
TH-GAL4/+ vs UAS-shi^{ts1}/+	0.2172	0.9743
TH-GAL4/UAS-shi^{ts1} vs *TH*-GAL4/+	4.8254	< 0.001
TH-GAL4/UAS-shi^{ts1} vs UAS-shi^{ts1}	4.2847	< 0.001

Table S4. Statistical comparison between genotypes in the CI of naive males, related to Figure 3B

Genotypes	Steel–Dwass test (q)	P
TH-GAL4/+ vs UAS-shi^{ts1}/+	0.2094	0.9761
TH-GAL4/UAS-shi^{ts1} vs *TH*-GAL4/+	4.3553	< 0.001
TH-GAL4/UAS-shi^{ts1} vs UAS-shi^{ts1}	4.3524	< 0.001

Table S5. Statistical comparison between genotypes in the CI of naive males, related to Figure 4A

Genotypes	Steel–Dwass test (q)	P
CS vs $Dop1R1^{KOGAL4}$ hetero	0.5977	0.9989
CS vs $Dop1R1^{KOGAL4}$ homo	0.9256	0.9837
$Dop1R1^{KOGAL4}$ hetero vs $Dop1R1^{KOGAL4}$ homo	0.2355	1.0000
CS vs $Dop1R2^{KOGAL4}$ hetero	0.3885	0.9999
CS vs $Dop1R2^{KOGAL4}$ homo	2.5285	0.1833
$Dop1R2^{KOGAL4}$ hetero vs $Dop1R2^{KOGAL4}$ homo	2.7682	0.1030
CS vs $Dop2R^{KOGAL4}$ hemi	0.0550	1.0000
CS vs $DopEcR^{KOGAL4}$ hetero	2.0118	0.4737
CS vs $DopEcR^{KOGAL4}$ homo	1.6204	0.7381
$DopEcR^{KOGAL4}$ hetero vs $DopEcR^{KOGAL4}$ homo	0.3647	1.0000

Table S6. Statistical comparison between genotypes in the CI of naive males, related to Figure 4B

Genotypes	Steel–Dwass test (q)	P
CS vs $Dop1R1^{KOGAL4}$ hetero	0.8286	0.9915
CS vs $Dop1R1^{KOGAL4}$ homo	2.8319	0.0872
$Dop1R1^{KOGAL4}$ hetero vs $Dop1R1^{KOGAL4}$ homo	3.1393	0.0361
CS vs $Dop1R2^{KOGAL4}$ hetero	1.7690	0.6413
CS vs $Dop1R2^{KOGAL4}$ homo	3.7424	0.0045
$Dop1R2^{KOGAL4}$ hetero vs $Dop1R2^{KOGAL4}$ homo	4.0572	0.0013
CS vs $Dop2R^{KOGAL4}$ hemi	1.8914	0.5568
CS vs $DopEcR^{KOGAL4}$ hetero	0.1504	1.0000
CS vs $DopEcR^{KOGAL4}$ homo	3.8615	0.0028
$DopEcR^{KOGAL4}$ hetero vs $DopEcR^{KOGAL4}$ homo	3.1900	0.0308

Table S7. Statistical comparison between genotypes in the CI of naive males, related to Figure 4D

Genotypes	Steel–Dwass test (q)	P
R13F02-GAL4/+ vs UAS-*Dop1R1* RNAi, UAS-*Dicer2*/+	2.5468	0.1428
R13F02-GAL4/UAS-*Dop1R1* RNAi, UAS-*Dicer2* vs *R13F02*-GAL4/+	1.8803	0.4934
R13F02-GAL4/UAS-*Dop1R1* RNAi, *UAS-Dicer2* vs UAS-*Dop1R1* RNAi, UAS-*Dicer2*/+	0.6559	0.9948
R13F02-GAL4/+ vs UAS-*Dop1R2* RNAi/+	1.0514	0.9419
R13F02-GAL4/UAS-*Dop1R2* RNAi vs *R13F02*-GAL4/+	0.9986	0.9545
R13F02-GAL4/UAS-*Dop1R2* RNAi vs UAS-*Dop1R2* RNAi/+	0.2080	1.0000
R13F02-GAL4/+ vs UAS-*Dop2R* RNAi, UAS-*Dicer2*/+	2.2425	0.2727
R13F02-GAL4/UAS-*Dop2R* RNAi, UAS-*Dicer2* vs *R13F02*-GAL4/+	1.2739	0.8640
R13F02-GAL4/UAS-*Dop2R* RNAi, *UAS-Dicer2* vs UAS-*Dop2R* RNAi, UAS-*Dicer2*/+	3.1476	0.0275

Table S8. Statistical comparison between genotypes in the CI of naive males, related to Figure 5A

Genotypes	Steel–Dwass test (q)	P
UAS-shi^{ts1}/+ vs *R58E02*-GAL4/+	1.0768	0.9350
R58E02-GAL4/UAS-shi^{ts1} vs UAS-shi^{ts1}/+	1.5331	0.7248
R58E02-GAL4/UAS-shi^{ts1} vs *R58E02*-GAL4/+	2.4130	0.1929
UAS-shi^{ts1}/+ vs *MB504B*-GAL4/+	1.8681	0.5016
MB504B-GAL4/UAS-shi^{ts1} vs UAS-shi^{ts1}/+	1.2939	0.8550
MB504B-GAL4/UAS-shi^{ts1} vs *MB504B*-GAL4/+	0.5191	0.9986
UAS-shi^{ts1}/+ vs *NP5945*-GAL4/+	2.4985	0.1596
NP5945-GAL4/UAS-shi^{ts1} vs UAS-shi^{ts1}/+	1.0876	0.9320
NP5945-GAL4/UAS-shi^{ts1} vs *NP5945*-GAL4/+	1.1630	0.9079

Table S9. Statistical comparison between genotypes in the CI of naive males, related to Figure 5B

Genotypes	Steel–Dwass test (q)	P
UAS-*shi*^{ts1}/+ vs *R58E02*-GAL4/+	0.3205	0.9999
R58E02-GAL4/UAS-*shi*^{ts1} vs UAS-*shi*^{ts1}/+	3.1953	0.0236
R58E02-GAL4/UAS-*shi*^{ts1} vs *R58E02*-GAL4/+	3.2711	0.0185
UAS-*shi*^{ts1}/+ vs *MB504B*-GAL4/+	2.3081	0.2398
MB504B-GAL4/UAS-*shi*^{ts1} vs UAS-*shi*^{ts1}/+	1.9420	0.4521
MB504B-GAL4/UAS-*shi*^{ts1} vs *MB504B*-GAL4/+	0.6173	0.9963
UAS-*shi*^{ts1}/+ vs *NP5945*-GAL4/+	1.0250	0.9484
NP5945-GAL4/UAS-*shi*^{ts1} vs UAS-*shi*^{ts1}/+	0.6189	0.9962
NP5945-GAL4/UAS-*shi*^{ts1} vs *NP5945*-GAL4/+	0.5928	0.9970
